# Strawberry (cv. *Romina*) Methanolic Extract and Anthocyanin-Enriched Fraction Improve Lipid Profile and Antioxidant Status in HepG2 Cells

**DOI:** 10.3390/ijms18061149

**Published:** 2017-05-28

**Authors:** Tamara Y. Forbes-Hernández, Massimiliano Gasparrini, Sadia Afrin, Danila Cianciosi, Ana M. González-Paramás, Celestino Santos-Buelga, Bruno Mezzetti, José L. Quiles, Maurizio Battino, Francesca Giampieri, Stefano Bompadre

**Affiliations:** 1 Dipartimento di Scienze Cliniche Specialistiche ed Odontostomatologiche (DISCO)-Sez. Biochimica, Facoltà di Medicina, Università Politecnica delle Marche, 60131 Ancona, Italy; tamara.forbe@gmail.com (T.Y.F.-H.); gaspamassi@libero.it (M.G.); dolla.bihs@gmail.com (S.A.); danila.cianciosi@gmail.com (D.C.); m.a.battino@univpm.it (M.B.); 2 Área de Nutrición y Salud, Universidad Internacional Iberoamericana (UNINI), Campeche C.P. 24040, Mexico; 3 Grupo de Investigación en Polifenoles (GIP-USAL), Faculty of Pharmacy, Campus Miguel de Unamuno, Salamanca University, Salamanca E-37007, Spain; paramas@usal.es (A.M.G.-P.); csb@usal.es (C.S.-B.); 4 Dipartimento di Scienze Agrarie, Alimentari e Ambientali, Università Politecnica delle Marche, 60131 Ancona, Italy; b.mezzetti@univpm.it; 5 Department of Physiology, Institute of Nutrition and Food Technology “José Mataix”, Biomedical Research Centre, University of Granada, Granada C.P. 18000, Spain; jlquiles@ugr.es; 6 Centre for Nutrition & Health, Universidad Europea del Atlantico (UEA), Santander 39011, Spain; 7 Dipartimento di Scienze Biomediche e Sanità Pubblica, Università Politecnica delle Marche, 60131 Ancona, Italy

**Keywords:** strawberry, anthocyanins, hypocholesterolemic, intracellular reactive oxygen species  diminution, antioxidant

## Abstract

Dyslipidemia and oxidation of low density lipoproteins (LDL) are recognized as critical factors in the development of atherosclerosis. Healthy dietary patterns, with abundant fruit and vegetable consumption, may prevent the onset of these risk factors due to the presence of phytochemical compounds. Strawberries are known for their high content of polyphenols; among them, flavonoids are the major constituents, and it is presumed that they are responsible for the biological activity of the fruit. Nevertheless, there are only a few studies that actually evaluate the effects of different fractions isolated from strawberries. In order to assess the effects of two different strawberry extracts (whole methanolic extract/anthocyanin-enriched fraction) on the lipid profile and antioxidant status in human hepatocellular carcinoma (HepG2) cells, the triglycerides and LDL-cholesterol content, lipid peroxidation, intracellular reactive oxygen species (ROS) content and antioxidant enzymes’ activity on cell lysates were determined. Results demonstrated that both strawberry extracts not only improved the lipid metabolism by decreasing triglycerides and LDL-cholesterol contents, but also improved the redox state of HepG2 cells by modulating thiobarbituric acid-reactive substances production, antioxidant enzyme activity and ROS generation. The observed effects were more pronounced for the anthocyanin-enriched fraction.

## 1. Introduction

Fruit and vegetable consumption has been recognized as an important dietary factor that could reduce the development of many chronic diseases [1,2], including metabolic syndrome [3,4,5], obesity [6], diabetes [7] and cardiovascular incidents, such as hypertension [8,9], coronary heart disease, stroke [10] and myocardial infarction [8], which are currently the leading causes of death in the world [11,12].

Although the mechanisms underlying the positive effects of fruits and vegetables on cardiovascular risks’ reduction are not completely clear [8,10], some of their constituents such as fiber, potassium, magnesium, folate and mainly polyphenols, especially flavonoids, appear to be responsible for them [9]. The principal mechanisms proposed for dietary flavonoids regarding the protection against cardiovascular diseases (CVDs) include improvement of the endothelial function through the reduction of low density lipoproteins’ (LDL) oxidation [13], the inhibition of endothelial NADPH oxidase and modulation of nitric oxide synthase activity/expression [14], the diminution of inflammatory biomarkers [15] and the enhancement of lipid profile and the redox status [13].

Rangel-Huerta et al. [15] summarized how certain foods or individual polyphenols isolated from these may interfere with the mentioned mechanisms. For example, grape ethanolic extract, resveratrol, curcumin or isoflavones decrease total cholesterol and triglyceride content and decrease oxidized LDL; while red wine, green/black tea and epillocatechin gallate-supplemented olive oil decrease inflammatory biomarkers such as necrosis factor α, plasminogen activator inhibitor 1 and interleukin (IL)-6/IL-10.

In the particular case of LDL oxidation and altered lipid profile, these are considered critical factors in the development of atherosclerosis, since primary events of atherogenesis begin with the uptake of oxidized LDL by endothelial cells or macrophages, which leads to the accumulation of foam cells within the atherosclerotic plaques and the formation of fatty streaks. These events cause cytotoxicity and vascular dysfunction [16]. When endothelial dysfunction occurs, it induces inflammation, oxidative stress, abnormal growth, immune dysfunction, vasoconstriction, increased permeability, thrombosis and ultimately atherosclerosis [17].

Scientific evidence indicates that dietary antioxidants reduce the clinical manifestations of CVDs by reducing LDL oxidation and subsequent cellular response to oxidized LDL [18]. In that sense, strawberries present a relevant antioxidant capacity, higher (from 2- to 11-fold) than apples, peaches, pears, grapes, tomatoes, oranges or kiwifruit [19]. They are one of the richest dietary sources of phytochemicals [20,21], mainly represented by flavonoids (mainly anthocyanins, with flavonols and flavanols providing a minor contribution), followed by hydrolysable tannins (ellagitannins and gallotannins) and phenolic acids (hydroxybenzoic acids and hydroxycinnamic acids). These polyphenols are known for their antioxidant and anti-atherosclerotic properties [22]; however, it is not well understood which specific groups are responsible for the mentioned effects. Some authors suggest that the combination of antioxidant micronutrients and polyphenol compounds may play a synergistic and cumulative role in health promotion [23].

In the particular case of the *Romina* strawberry variety (AN99.78.51), which is a new cultivar released in 2011 as a result of the breeding program of Marche Polytechnic University (UNIVPM, Ancona, Italy), there is a growing interest due to its valuable agronomic characteristics associated with a high adaptability to non-fumigated soil and to open field cultivation in climatic conditions from the mid-Adriatic to the center-north of Europe, as well as to its resistance to the major strawberry diseases. It is also of interest for producers and consumers for its early ripening time and its nutritional quality.

According to Capocasa et al. [24], *Romina* fruit quality is recognized for its higher content of soluble solids (SS) (7.7° Brix) combined with low total acidity (10.1 mEq NaOH/100 g), which confer to the fruit a very high perception of sweetness, a well-appreciated characteristic for the consumer. To this important sensorial trait, *Romina* fruit also combines a high content of anthocyanins and an elevated antioxidant capacity. In addition, its contents of vitamin C, folic acid and flavonols are good, so it is expected that its health benefits are high.

The main objective of the present work was to evaluate the effects of two different *Romina* strawberry extracts (whole methanolic extract/anthocyanin-enriched fraction) on the lipid profile and antioxidant status in HepG2 cells.

## 2. Results

### 2.1. Characterization of Strawberry Extracts

The results of the phytochemical and antioxidant characterization of strawberry extracts are shown in Table 1. The anthocyanin-enriched fraction revealed higher values of total polyphenol content (TPC) (531.99 ± 2.01 mg GAeq/g dried weight (DW)), total flavonoids content (TFC) (247.22 ± 2.56 mg CATeq/g DW) and total antioxidant capacity (TAC) (4400 ± 11.43 µmol Txeq/g DW, by ferric-reducing antioxidant power (FRAP) assay; 1590 ± 3.54 µmol Txeq/g DW, by 2,2-diphenyl-1-picrylhydrazyl (DPPH) free radical method; 167.58 ± 2.64 µmol Txeq/g DW, by trolox equivalent antioxidant capacity (TEAC) assay compared to the whole methanolic extract. These results were partially expected taking into account that the anthocyanin-enriched extract is a purified fraction (in order to increase the anthocyanin concentration), while the whole methanolic extract may contain not only phytochemicals, but also other compounds, such as sugars, vitamins and other types of compounds [25] that contribute to the total weight, but not to the evaluated parameters, and may also interfere with the action of phytochemicals. 

However, the marked difference between the extracts regarding the antioxidant capacity was really interesting, considering that the *Romina* strawberry variety contains good levels of vitamin C (38.5 mg/100 g fresh weight (FW)) [24], which, in addition to polyphenols, is one of the major contributors to this parameter and whose concentrations in the whole methanolic extract should be higher than in the anthocyanin-enriched fraction, taking into account that it is eliminated during the purification/concentration process.

The anthocyanin family is definitely the major component of both fractions, representing approximately 86% to 94% of the total phenolic compound identified through the high performance liquid chromatography (HPLC) analysis (Table 2). These results are in accordance with the data reported by He et al. [26], who evaluate different methods for anthocyanin isolation from fruits and vegetables, obtaining anthocyanins with purity over 83% when the traditional C18 method of solid phase extraction (SPE) was employed.

Other phenolic compounds (ellagic acid derivatives and flavonols/dihydroflavonols) have also been identified in both fractions even though in a small quantity.

The main anthocyanin identified, pelargonidin 3-*O*-glucoside, represented 80% of the total anthocyanins, which is in correspondence with the principal anthocyanin reported for strawberries. In the fresh fruit pelargonidin-3-*O*-glucoside, cyanidin-3-*O*-glucoside and pelargonidin-3-*O*-rutinoside are recognized as the major compounds of this family [27,28,29], contributing on average to 41% of the TPC [28]. The total concentration of this group of phenolic compounds varies from 8.5 to 65.9 mg/100 g fresh weight (FW) [27,28,29] depending on strawberry variety, climactic conditions and post-harvest handling procedures, among other factors. The following two groups that have a greater contribution to the TPC in strawberry are flavonols and ellagitannins [27,28].

### 2.2. Effects of Strawberry Extracts on HepG2 Cell Viability

To evaluate the possible cytotoxic effects of strawberry fractions in HepG2 cells, an MTT (3-(4,5-dimethylthiazol-2-yl)-2,5-diphenyltetrazolium bromide) assay was performed. Cells were incubated with extensive concentration ranges of both the whole methanolic extract and the anthocyanin fraction for 24 h

**Figure 1:**
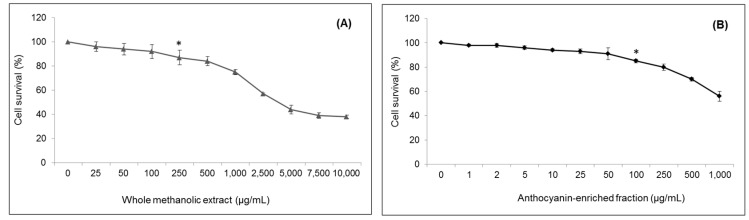
Viability of HepG2 cells after treatment with strawberry whole methanolic extract (**A**) and the anthocyanin-enriched fraction (**B**). Cells were treated with the indicated concentration of strawberry extracts for 24 h. Values are expressed as the mean ± SD of three independent experiments (*n* = 3). The asterisk indicates the concentrations from which significant differences (*p* < 0.05) were observed compared to the control.

After treatment with the whole methanolic extract, cell viability was not significantly affected (*p* < 0.05) at concentrations up to 100 µg/mL, but significant cytotoxicity was revealed at higher concentrations. Likewise, the anthocyanin-enriched fraction at concentrations lower than 50 µg/mL did not cause significant (*p* < 0.05) cell death compared to the control (Figure 1). Hence, the concentrations of 10, 50 and 100 µg/mL of whole methanolic extract and 5, 10 and 50 µg/mL of the anthocyanin fraction, corresponding to approximately 98, 95 and 91% of viable cells, respectively, were used in subsequent experiments.

### 2.3. Effects of Strawberry Extracts on Lipid Profile and Lipid Peroxidation

**Figure 2:**
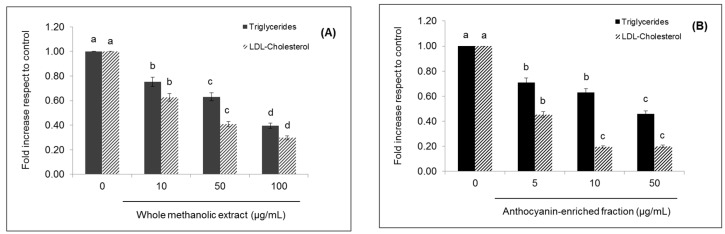
Effects of strawberry whole methanolic extract (**A**) and the anthocyanin-enriched fraction (**B**) on triglycerides and LDL-cholesterol content in HepG2 cells. Cells were treated with the indicated concentration of strawberry extracts for 24 h. The concentration of 0 µg/mL corresponds to the control (untreated cells). Values are expressed as the mean ± SD (*n* = 3) of three independent experiments. Columns belonging to the same set of data with different superscript letters are significantly different (*p* < 0.05).

As shown in Figure 2, strawberry whole methanolic extract significantly (*p* < 0.05) decreased triglyceride levels in a dose-dependent manner up to 0.40-fold compared to the control when applied at the highest concentration evaluated (100 µg/mL). In the case of the anthocyanin-enriched fraction, no significant differences (*p* < 0.05) were observed between the two lowest concentrations (5 and 10 µg/mL), but in all cases, the triglyceride level diminution was significant (*p* < 0.05) compared to the untreated cells, reaching 0.46-fold when applied at 50 µg/mL. 

LDL-cholesterol levels were also significantly (*p* < 0.05) decreased by both strawberry extracts (Figure 2). Maximum concentrations of the whole methanolic extract/anthocyanin-enriched fraction caused a diminution of this indicator up to 0.30- and 0.19-fold compared to the control, respectively.

Although for both extracts, higher effects were observed at the higher concentrations used, already at the lower concentration, the decrease in the content of triglycerides and LDL-cholesterol was significant (*p* < 0.05) compared to the control.

**Figure 3:**
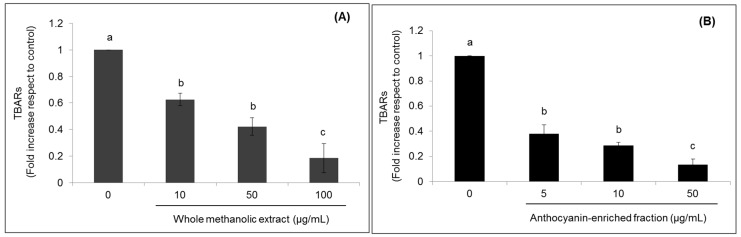
Effects of strawberry whole methanolic extract (**A**) and the anthocyanin-enriched fraction (**B**) on lipid peroxidation in HepG2 cells. Cells were treated with the indicated concentration of strawberry extracts for 24 h. The concentration of 0 µg/mL corresponds to the control (untreated cells). Values are expressed as the mean ± SD of three independent experiments (*n* = 3). Columns belonging to the same set of data with different superscript letters are significantly different (*p* < 0.05).

Furthermore, strawberry extracts not only improved the lipid profile by lowering LDL-cholesterol and triglyceride levels, but also reduced lipid peroxidation, as shown in Figure 3. Certain diagnostic tests are available for the quantification of the end-products of lipid peroxidation, with the thiobarbituric acid-reactive substance (TBARs) assay as the most commonly used. The highest concentrations of both strawberry extracts significantly (*p* < 0.05) decreased the TBARs levels up to 0.18-fold compared to untreated cells.

### 2.4. Effects of Strawberry Extracts on Intracellular Production of Reactive Oxygen Species and Antioxidant Enzyme Activity

**Figure 4:**
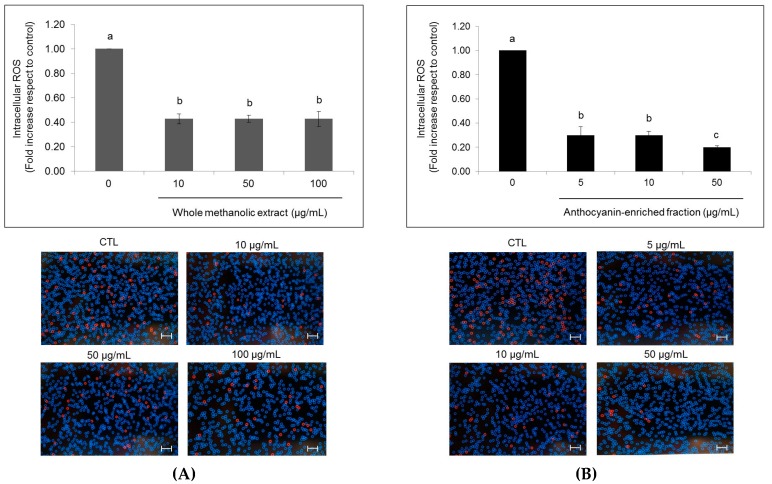
Effects of strawberry whole methanolic extract (**A**) and the anthocyanin-enriched fraction (**B**) on intracellular reactive oxygen species (ROS) production in HepG2 cells. Cells were treated with the indicated concentration of strawberry extracts for 24 h. The concentration of 0 µg/mL corresponds to the control (untreated cells). Scale bar, 50 µm. Representative images of intracellular ROS quantification by the Tali^®^ Image-Based Cytometer (Thermo Fisher Scientific, Milan, Italy) are shown following the graphs (stressed cells appear red). Values are expressed as the mean ± SD of three independent experiments (*n* = 3). Columns belonging to the same set of data with different superscript letters are significantly different (*p* < 0.05).

Regarding intracellular reactive oxygen species (ROS) production, a significant diminution (*p* < 0.05) was outlined in cells supplemented with both strawberry extracts (Figure 4). After 24 h of treatment, whole methanolic extract caused a decrease up to 0.43-fold compared to untreated cells independently of the concentrations in which it was applied. Meanwhile, the anthocyanin-enriched fraction caused a diminution of 0.30- and 0.20-fold when it was applied at 5 to 10 and 50 µg/mL, respectively.

**Figure 5:**
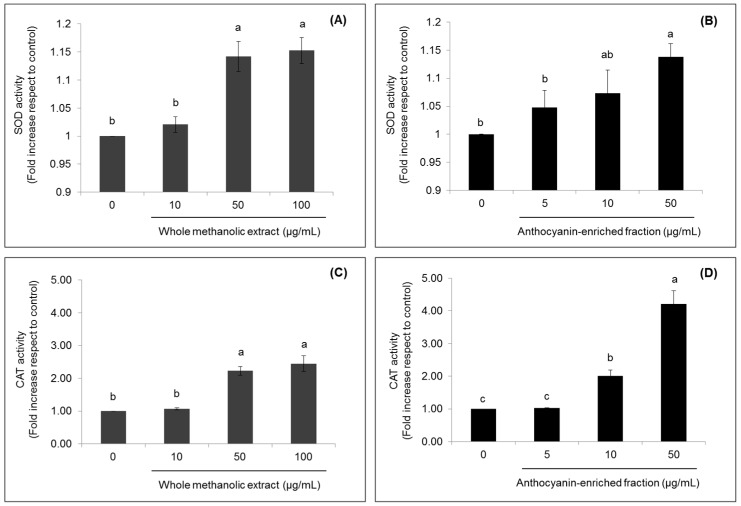
Effects of strawberry whole methanolic extract (**A**,**C**) and the anthocyanin-enriched fraction (**B**,**D**) on superoxide dismutase (SOD) and catalase (CAT) activities in HepG2 cells. Cells were treated with the indicated concentrations of strawberry extracts for 24 h. The concentration of 0 µg/mL corresponds to the control (untreated cells). Values are expressed as the mean ± SD of three independent experiments (*n* = 3). Columns belonging to the same set of data with different superscript letters are significantly different (*p* < 0.05).

Closely related to the intracellular ROS production and lipid peroxidation is the state of the antioxidant defense systems. Hence, the activity of the antioxidant enzymes superoxide dismutase (SOD) and catalase (CAT) were also evaluated. SOD activity significantly increased (*p* < 0.05) up to 1.15-fold compared to the control, after treatment with dried methanolic extract (50 and 100 µg/mL) (Figure 5A) or the anthocyanin fraction at 50 µg/mL (Figure 5B). The lowest concentration evaluated for both extracts (5 or 10 µg/mL) did not cause significant (*p* < 0.05) effects compared to untreated cells.

Furthermore, the CAT activity significantly increased (*p* < 0.05) after treatment with strawberry extracts, even in a greater proportion compared to the effects observed for SOD. In cells treated with 50 and 100 µg/mL of whole methanolic extract, CAT activity was respectively 2.22- and 2.45-fold higher compared to the control, while in those cells treated with 10 and 50 µg/mL of the anthocyanin-enriched fraction, it increased up to 2.01- and 4.21-fold compared to untreated cells, respectively; in this case, the minimal concentrations evaluated of both extracts did not have a significant (*p* < 0.05) effect on the enzyme activity.

Comparing the effects that were caused by both strawberry extracts when applied at the same concentrations (10 and 50 µg/mL), it can be noticed that the anthocyanin-enriched fraction was more effective in almost all cases (Table 3), excluding the effect in SOD activity where no significant differences were observed (*p* < 0.05).

Interestingly, the major difference between the two extracts was observed in the induction of the other antioxidant enzyme activity, CAT. The anthocyanin-enriched extract caused an increase in CAT activity 94% and 199% higher than the whole methanolic extract when applied at 10 and 50 µg/mL, respectively.

## 3. Discussion

In the present work, we demonstrated that two different strawberry fractions (whole methanolic extract and the anthocyanin-enriched fraction) decreased total cholesterol and triglyceride content, lipid peroxidation, intracellular ROS production and increased antioxidant enzymes’ activity, although to different extents.

For all of the evaluated parameters, the anthocyanin-enriched fraction resulted in being more effective than the whole methanolic extract, since lower concentrations were needed to obtain similar effects, or in other words, when they were applied at equal concentrations, the effects for the anthocyanin fraction were more noticeable.

Although there are only a few studies evaluating the biological effects of anthocyanins isolated from strawberries, the hypothesis that these compounds are mainly responsible for the biological activities of this fruit has been sustained by some authors. For example, Prior et al. [30] demonstrated that supplementation of drinking water with purified anthocyanins, but not whole strawberries, altered the development of obesity in mice. Likewise, Fotschki et al. [31] confirmed that the addition of anthocyanins in a strawberry polyphenolic extract enhanced the positive effects of diets with fructooligosaccharides (FOS) in the rat cecal environment.

Other results from human studies evaluating the effects of anthocyanins isolated from berries on human health demonstrated that supplementation with 320 mg/day of purified anthocyanins from bilberries (*Vaccinium myrtillus*) and black currants (*Ribes nigrum*) for 12 weeks decreased LDL-cholesterol levels (up to 13.6%) in dyslipidemic subjects [32], while the ingestion of 500 mg/day of elderberry extract for the same period of time resulted in being ineffective in improving biomarkers of CVDs’ risk in healthy postmenopausal women [33].

It has been suggested that anthocyanins are the major contributors to the TAC of berries [34], nevertheless, it would be interesting to further analyze if they are also mainly responsible for other biological activities reported for strawberries, how the structure could affect these properties and what are the potential synergistic interactions among the anthocyanins. It would also be interesting to evaluate the biological properties of the metabolites resulting from the digestion of these compounds, since it has been demonstrated that anthocyanin bioavailability is relatively low (relative urinary excretions, ranging from 0.004% to 0.1% of the intake) compared to other polyphenols [29,35].

For example, in a study conducted by Banaszewski et al. [36], the maximal concentrations of pelargonidin-3-*O*-glucoside (the most abundant metabolite identified) achieved in the plasma of healthy volunteers after 148 ± 31 min of having consumed four beverages containing 0, 10, 20 and 40 g of strawberry powder were 0, 93.4 ± 21.9, 166.5 ± 16.2 and 226.7 ± 36.7 nmol/L, respectively. In addition, some authors have demonstrated that also the timing of intake may influence anthocyanin bioavailability and therefore their health promoting effects. In that sense, Sandhu et al. [29] have reported that plasma concentration of pelargonidin-3-*O*-glucoside increased ≈ 66% when a strawberry drink was consumed in a fasted state compared to the fed state. 

In general, although many researchers are investigating the potential health benefits of fruit anthocyanins, in most cases, they use crude extracts without eliminating potentially bioactive impurities, which could have biological effects, creating interference in the bioassays. Access to high-purity anthocyanin extracts is essential for the validity of such research. 

Removal of undesirable compounds from anthocyanin extracts can also be of great importance for the food and nutraceutical industries. Sugars, phenolic compounds, amino acids and metals accelerate the degradation of anthocyanins, and therefore, high purity is desirable for improved stability [26]. 

However, these results may have a different interpretation, in the sense that greater quantities of strawberries are required to obtain the same amount of the anthocyanin fraction as the crude extract (in this work, 1 mg of whole methanolic extract was obtained from 23.31 mg of fresh fruit while 1 mg of the anthocyanin-enriched fraction was obtained from 211.90 mg fresh fruit), an aspect that must be taken into consideration during the formulation of nutraceutical products using strawberry as a bioactive ingredient.

Concerning the lipid peroxidation inhibition, our overall data were in correspondence with the results obtained by Giampieri et al. [37] and Alvarez-Suarez et al. [38] who reported that strawberry polyphenols are able to suppress lipid peroxidation in vitro and in vivo, respectively.

Lipid peroxidation is a complex process that involves the formation and propagation of lipid radicals, the uptake of oxygen, a rearrangement of the double bonds in unsaturated lipids and the eventual destruction of membrane lipids, with the production of a variety of breakdown products. Its inhibition occurs through enzymatic reactions or through free radical scavenging by antioxidants. An increased concentration of end products of lipid peroxidation is the evidence most frequently quoted for the involvement of free radicals in human disease. Actually, lipid peroxidation is considered as one of the principal molecular mechanisms involved in oxidative damage to cell structures and in the toxicity process that leads to cell death [39]. In atherosclerosis and in worsening the initial tissue injury caused by ischemic or traumatic brain damage, lipid peroxidation seems to play an important pathological role. Hence, its inhibition/diminution is crucial to preventing these diseases, and for that reason, the role of antioxidants has received extensive attention. 

Likewise, the diminution of intracellular ROS production by strawberry methanolic extract has also been reported by Giampieri et al. [40,41] in human dermal fibroblasts stressed with 2,2′-azobis(2-amidinopropane) dihydrochloride (AAPH) or hydrogen peroxide (H_2_O_2_), respectively; but there is no information about strawberry anthocyanin fraction effects. The clinical implications of elevated ROS production can be severe and become a major cause of molecular injury leading to cell aging and to age-related degenerative diseases. In this regard, the liver is particularly susceptible to toxic and oxidative insults.

Regarding antioxidant enzyme activities, the obtained results were in agreement with the observed decrease in lipid peroxidation and intracellular ROS production since, as mentioned above, these three aspects are closely related. Changes in the activity of antioxidant enzymes can be considered as biomarkers of the antioxidant response. SOD, a free radical scavenger, is one of the major defenses against the oxidizing effect of the superoxide radical. It could protect cells from the toxicity of superoxide radicals by transforming them to H_2_O_2_, which is subsequently converted by CAT in water and oxygen. In HepG2 cells, the improvement of SOD and CAT activities has also been reported after treatment with some natural compounds, such as blueberry anthocyanidins [42], bioactive compounds of endophytic fungus from pigeon pea [43], cocoa polyphenolic extract [44] and resveratrol [45].

In our opinion, the observed effects of strawberry fractions may depend not only on their antioxidant capacity, but also on their ability to activate endogenous defense systems probably through the AMP-activated protein kinase (AMPK) pathway. AMPK could be involved in the antioxidant response of the organism through the activation of the nuclear related factor 2 (Nrf2) and consequently of some antioxidant responsive elements (AREs). Nrf2 is a basic leucine zipper transcription factor that upregulates ARE-driven detoxification and antioxidant genes. Since the expression of a wide array of antioxidant and detoxification genes is positively regulated by the ARE sequence, Nrf2 may serve as a master regulator of the ARE-driven cellular defense system against oxidative stress [46,47,48,49,50]. Since preliminary data of our group suggest that both strawberry fractions induce AMPK activity, this could be a possible mechanism of action; however, specific analyses are needed to confirm this hypothesis. In this sense, Yun et al. [49] demonstrated that AMPK activity plays an indispensable role in the operation of the ROS defense system by inducing the expression of the antioxidant enzymes (SOD and CAT) in response to resveratrol treatment in liver cells. 

## 4. Materials and Methods

### 4.1. Plant Material and Sampling Method

Strawberry fruits *Fragaria × Ananassa* (cv. *Romina*) were collected in the experimental fields of the Agricultural Faculty of the Università Politecnica delle Marche, Italy. Fruit samples were hand-picked on the same day-time in different weeks, corresponding to the ripening times of this variety, and were selected for homogeneity, avoiding unripe, wounded or shriveled samples. Within 2 h after harvest, whole strawberries were stored at −80 °C until the analyses’ execution.

### 4.2. Methanolic Extract Preparation

For methanolic extract preparation, 50 g of fruit were added to 100 mL of the extraction solution, consisting of methanol/Milli-Q water/concentrated formic acid (80:20:0.1, *v*/*v*/*v*), and were homogenized using an Ultraturrax T25 homogenizer (Janke & Kunkel, IKA Labortechnik, Staufen, Germany) at medium-high speed for 2 min. Extraction was maximized by stirring the suspension at 22× *g* (ARE Magnetic stirrer, VELP Scientifica, Usmate, Italy) for 2 h in the dark at room temperature. The mixture was then centrifuged at 2400× *g* for 15 min for two sequential times, in order to sediment solids. Supernatants were filtered through a 0.45-µm Minisart filter (PBI International, Milan, Italy) and transferred to a 5.0-mL amber glass. For subsequent experimental procedures, the methanolic extract was concentrated and dried through a rotary evaporator resulting in 1.6 g of dried material. The sample was stored in aliquots at –80 °C. 

### 4.3. Extraction of the Anthocyanins Fraction

The anthocyanin fraction was obtained as previously described by Alvarez-Suarez et al. [38]; 50 g of strawberries were homogenized in 100 mL of methanol containing 0.1% HCl, stirred overnight (22× *g*, ~14 h, 3 to 5 °C) and subsequently filtered through a Büchner funnel under vacuum. The solid residues were exhaustively washed with methanol, the number of times necessary to complete color extraction, and the filtrates obtained were centrifuged (4000× *g*, 15 min, 21 °C). All supernatants were mixed, dried through a rotary evaporator and re-suspended in 50 mL of water. Subsequently, aliquots (2 mL) of the aqueous phase were carefully charged into C18 SepPaks Vac 6cc cartridges (Waters, Milan, Italy) for solid phase extraction (SPE). Sugars and more polar substances were removed by passing 15 mL of ultrapure water, and anthocyanin pigments were further eluted with 5 mL of methanol/0.1% trifluoroacetic acid (95:5, *v*/*v*). The final methanolic extract was concentrated and dried again through a rotary evaporator resulting in 0.73 g of dried material. The sample was stored in aliquots at −80 °C. 

This kind of sample preparation permits removing sugar, organic acids and other water-soluble fruit constituents [25,51] and to obtain a highly concentrated anthocyanin solution, mainly non-polymerized anthocyanins [51,52,53]. Usually, the C18 cartridges do not adsorb the phenolic acids, which are collected in the first eluent (water,) while catechins and flavonols are eluted together with the non-polymerized anthocyanins [54].

### 4.4. Total Phenolic Content Determination

TPC of the strawberry fractions was determined using the Folin–Ciocalteu method, as modified by Slinkard and Singleton [55]. Briefly, 100 µL of water re-suspended strawberry fractions were added to 500 µL of Folin-Ciocalteu solution and kept at 4 °C in the dark. Next, the mixture was incubated for 1 to 8 min at room temperature, and 400 µL of 0.7 M sodium carbonate (Na_2_CO_3_) were added. The solution was incubated for 2 h at room temperature (~23 °C) in the dark, and the absorbance was read at 760 nm. Gallic acid solutions (0.5 to 3.0 mM) were used as the standard.

### 4.5. Total Flavonoid Content Determination

TFC was determined through a colorimetric method previously described by Jia et al. [56] and Dewanto et al. [57]. Briefly, 250 µL of water re-suspended strawberry fractions were mixed with 1.25 mL of Milli-Q water, followed by the addition of 75 µL of a 5% sodium nitrate (NaNO_2_) solution. After 6 min, 150 µL of a 10% aluminum chloride hexahydrate (AlCl_3_·6H_2_O) solution were added to the mixture and allowed to stand for 5 min. Then, 500 µL of 1M sodium hydroxide (NaOH) were added; the mixture was brought to 2.5 mL with Milli-Q water; and the absorbance was immediately read at 510 nm. (+)-Catechin solutions (0.0125 to 0.1 mg/mL) were used as the standard. 

### 4.6. Total Antioxidant Capacity Determination

For determination of TAC of the strawberry fractions, three different methods were employed: the FRAP assay, the DPPH free radical method and the TEAC assay.

The FRAP assay was carried out according to the protocol proposed by Deighton et al. [58], with slight modifications from the original method [59]. The antioxidant capacity of samples is determined by their ability to reduce ferric to ferrous ion. When iron is complexed with 2,4,6-tripyridyl-s-triazine (TPTZ) in sodium acetate solution at an acidic pH, its reduction results in a solution color change from pale rust to blue. The absorbance of the solution at 593 nm reflects the extent of the reduction.

The DPPH method was carried out according to the protocol proposed by Kumaran and Karunakaran [60]. It is based on the spectrophotometric measurement of the free radical DPPH reduction at 515 nm.

The TEAC assay was carried out according to the method proposed by Re et al. [61]. This method is based on the ability of antioxidant compounds to quench the 2,2′-azino-bis(3-ethylbenzothiazoline-6-sulfonic acid) (ABTS) radical with the consequent decrease in the absorbance values measured at 734 nm.

### 4.7. HPLC-MS Analysis

HPLC analyses were carried out in a Hewlett–Packard 1100 chromatograph (Agilent Technologies, Waldbronn, Germany) equipped with a quaternary pump and a diode array detector (DAD) coupled to an HP Chem Station(Santa Clara, CA, USA) (rev. A.05.04) data-processing station. The HPLC system was connected via the DAD cell outlet to an API 3200 Qtrap (Applied Biosystems, Darmstadt, Germany) mass spectrometer (MS) consisting of an ESI source and a triple quadrupole-ion trap mass analyzer, which was controlled by the Analyst 5.1 software (Waltham, MA, USA).

#### 4.7.1. Analysis of Anthocyanins

An AQUA^®^ (Phenomenex, Madrid, Spain) reverse phase C18 column (5 μm, 150 mm × 4.6 mm) thermostated at 35 °C was used. The solvents were: (A) 0.1% trifluoroacetic acid and (B) acetonitrile. The elution gradient established was: isocratic 10% B for 3 min, 10 to 15% B in 12 min, isocratic 15% B for 5 min, 15 to 18% B over 5 min, 18 to 30% B over 20 min, 30 to 35% B over 5 min and re-equilibration of the column to initial solvent conditions. The flow rate used was 0.5 mL/min. Double online detection was carried out in the DAD using 280 and 520 nm as preferred wavelengths and in the MS operated in the positive ion mode. Spectra were recorded between *m*/*z* 100 and *m*/*z* 1500. Zero grade air served as the nebulizer gas (40 psi) and as turbo gas (600 °C) for solvent drying (50 psi). Nitrogen served as the curtain (100 psi) and collision gas (high). Both quadrupoles were set at unit resolution, and the MS detector was programmed to perform a series of two consecutive analyses, a full scan of high sensitivity (enhanced MS (EMS)) and an enhanced product ion analysis (EPI) to obtain the fragmentation pattern of the parent ion. The EMS mode parameters were the following: ion spray voltage 5000 V, declustering potential (DP) 41 V, entrance potential (EP) 7.5 V and collision energy (CE) 10 V. EPI mode was applied using the following settings: DP 41 V, EP 7.5 V, CE 10 V and collision energy spread (CES) 0 V.

Compounds were identified by their retention time, UV-Vis spectra and mass spectra, as well as comparison with our data library and standards when available. The compounds were quantified from the areas of their chromatographic peaks recorded at 520 nm using pelargonidin-3-*O*-glucoside for calibration curves.

#### 4.7.2. Analysis of Flavonols and Other Phenolic Derivatives

An AQUA^®^ (Phenomenex) reverse phase C18 column (5 μm, 150 mm × 4.6 mm) thermostated at 35 °C was used. The solvents were: (A) 0.1% formic acid and (B) acetonitrile. The elution gradient established was isocratic 15% B for 5 min, 15 to 20% B over 5 min, 20 to 35% B over 10 min, 35 to 50% B over 10 min, 50 to 60% B over 5 min, isocratic 60% B for 5 min and re-equilibration the column to initial solvent conditions. The flow rate was 0.5 mL/min. Double online detection was carried out in the DAD at 280, 330 and 370 nm as preferred wavelengths and in the MS operated in the negative ion mode. Spectra were recorded between *m*/*z* 100 and *m*/*z* 1500. Zero grade air served as the nebulizer gas (30 psi) and as turbo gas (400 °C) for solvent drying (40 psi). Nitrogen served as the curtain (20 psi) and collision gas (medium). Both quadrupoles were set at unit resolution, and EMS and EPI analyses were also performed. The EMS parameters were: ion spray voltage 4500 V, DP −50 V, EP −6 V, CE −10 V and cell exit potential (CXP) −3 V; whereas EPI settings were: DP −50 V, EP −6 V, CE −25 V and CES 0 V.

Compounds were identified by their retention time, UV-Vis spectra and mass spectra, as well as the comparison with our data library and standards when available. The compounds were quantified from the areas of their chromatographic peaks recorded at 280 and 360 nm using ellagic acid, quercetin and kaempferol glucoside for the calibration curves constructed.

### 4.8. Cells Culture and Cells’ Lysates Preparation

HepG2 cells were kindly provided by the Biological Research Laboratory of Seville University (Seville, Spain) and were grown in Dulbecco’s Modified Eagle’s Medium (DMEM), supplemented with 10% fetal bovine serum (FBS), 100 IU/mL penicillin and 100 µg/mL streptomycin until 80 to 90% of confluence when sub-cultured. Cells were maintained in a HeraCell CO_2_ incubator at 37 °C with 5% CO_2_. After treatments with the strawberry fractions for the indicated periods, cells were lysed in the RIPA buffer (Sigma-Aldrich, Milan, Italy) for lipid profile, lipid peroxidation and enzyme activity determination. All of the analyses were conducted on cells between the 3rd and the 6th passage.

### 4.9. Cell Viability: MTT Assay

For cell viability assessment, HepG2 cells were seeded into 96-well plates at a density of 5 × 10^3^ cells/well and treated with different concentrations (from 0 to 1 mg/mL) of the strawberry fractions for 24, 48 and 72 h. Both the dried methanolic extract and the anthocyanin fraction were directly dissolved in the cell culture medium. After incubation, 30 µL of RPMI medium containing 2 mg/mL of MTT were added in each well, and cells were incubated for other 2 h at 37 °C. MTT solution was then discarded, and 100 µL of DMSO were added into each well to dissolve the formazan crystal. The level of colored formazan derivative was analyzed on a microplate reader (Thermo Scientific Multiskan^®^ EX, Monza, Italy) at a wavelength of 590 nm [62,63]. The viable cells were directly proportional to the formazan production.

### 4.10. Determination of Triglycerides and LDL-Cholesterol Content

LDL-cholesterol and triglyceride contents were determined by enzymatic colorimetric kits (Spinreact, St. Esteve d’en Bas, Girona, Spain) using a microplate reader (Thermo Scientific, Multiskan^®^ EX, Monza, Italy) coupled to an Ascent software (Thermo LabSystems Oy, Version 2.6, Milan, Italy).

### 4.11. Determination of Lipid Peroxidation: TBARs Assay

Lipid peroxidation was measured by the TBAR_S_ assay according to a standardized method proposed by Ohkawa et al. [64]. Briefly, 300 µL of cellular lysate were mixed with the thiobarbituric acid (TBA) reagent (TBA, 0.37% in 0.2 M HCl) and 15 % trichloroacetic acid (TCA) and heated at 95 °C for 20 min. Then, the mixture was cooled, centrifuged at 1200× *g* for 15 min at 4 °C, and the supernatant absorbance was measured at 532 nm. 

### 4.12. Assessment of Intracellular ROS Production by the Tali^®^ Image-Based Cytometer

Determination of intracellular ROS levels was performed using the CellROX^®^ Oxidative Stress Kit (Invitrogen TM, Life Technologies, Milan, Italy) according to the manufacturer’s instructions. Briefly, cells were seeded in 6-well plates at a density of 1.5 × 10^5^ cells/well and treated with different concentrations of the strawberry fractions for 24 h. The concentrations used for each fraction were chosen according to the MTT viability assay ensuring a vitality greater than 90%. After treatment, cells were detached by trypsinization and centrifuged at 556× *g* for 10 min at 4 °C. The supernatant was discarded, re-suspending the cellular pellet in 1 mL of complete medium. Then, CellROX^®^ Orange Reagent was added at a final concentration of 5 µM, and samples were incubated for 30 min at 37 °C, centrifuged once to remove medium and dye excesses and re-suspended again in PBS. After labeling with CellROX^®^ Orange Reagent, cells were analyzed with the Tali^®^ Image-Based cytometer (Thermo Fisher Scientific, Milan, Italy).

### 4.13. Evaluation of Antioxidant Enzymes Activity

SOD activity was assayed according to the method proposed by Kakkar et al. [65]. The method is based on the dismutation of the superoxide radical to oxygen and H_2_O_2_. Briefly, the assay mixture contained 1.2 mL of 0.025 M sodium pyrophosphate buffer, pH 8.3, 100 µL of 186 µM phenazine methosulfate, 300 µL of 300 µM nitroblue tetrazolium, 190 µL mL of PBS, 10 µL of the cellular lysate and 1 mL of water, for a total volume of 2.8 mL. The reaction was initiated by the addition of 10 µL of NADH, and the mixture was incubated at 30 °C for 90 s and arrested by the addition of 1.0 mL of glacial acetic acid. The reaction mixture was then shaken with 2 mL of n-butanol, allowed to stand for 10 min and centrifuged at 1300× *g* for another 10 min. The intensity of the chromogen in the butanol layer was measured at 540 nm in a microplate reader (Thermo Scientific, Multiskan^®^ EX, Monza, Italy).

CAT activity was assayed according to the method proposed by Aebi [66]. The method is based on the decomposition of H_2_O_2_ by the action of the enzyme. Briefly, the assay mixture consisted of 990 µL of sodium phosphate buffer (50 mM) pH 7, 500 µL of H_2_O_2_ (30%) and 10 µL of the cellular lysate. The decrease in absorbance due to H_2_O_2_ degradation was monitored at 240 nm past 10 to 70 s of reaction. 

## 5. Conclusions

Our results demonstrated that strawberry fractions not only improved the lipid metabolism by decreasing triglycerides and LDL-cholesterol contents, but also improved the redox state of HepG2 cells by modulating TBARs production, antioxidant enzyme activity and ROS generation. The anthocyanin-enriched fraction resulted in being more effective than the whole methanolic extract for almost all of the evaluated parameters. Further studies must be conducted in order to confirm these findings also in humans or animal models.

## Figures and Tables

**Table 1:** Phytochemical characterization and antioxidant capacity of the extracts.

Parameters/Fractions	Whole Methanolic Extract	Anthocyanin-Enriched Fraction
**Phytochemical Characterization**		
Total polyphenols (mg GAeq/g DW)	23.44 ± 0.22 ^b^	531.99 ± 2.01 ^a^
Flavonoids (mg CATeq/g DW)	5.21 ± 0.29 ^b^	247.22 ± 2.56 ^a^
**TAC (µmol Txeq/g DW)**		
FRAP	168.25 ± 3.95 ^b^	4400 ± 11.43 ^a^
DPPH	30.29 ± 0.18 ^b^	1590 ± 3.54 ^a^
TEAC	35.51 ± 0.06 ^b^	167.58 ± 2.64 ^a^

mg GAeq/g DW: mg of gallic acid equivalent/g of the dried weight (DW) of the fraction. mg CATeq/g DW: mg of catechin equivalent/g of the dried weight (DW) of the fraction. µmol Txeq/g DW: µmol of Trolox equivalent/g of the dried weight (DW) of the fraction. FRAP: ferric-reducing antioxidant power assay. DPPH: 2,2-diphenyl-1-picrylhydrazyl free radical method. TEAC: trolox equivalent antioxidant capacity assay; TAC, total antioxidant capacity. Different superscripts letter for each column indicated significant differences (*p* < 0.05).

**Table 2:** Identification and quantification of the main phenolic compounds present in both fractions.

**Peak Number**	***λ*_max_ (nm)**	**[M]^+^ (*m*/*z*)**	**MS^2^ 2^nd^ Stage of MS Spectrometry**	**Tentative Identification**	**Whole Methanolic Extract**	**Anthocyanin-Enriched Fraction**
**Anthocyanins (Expressed as Pg 3-*O*-glc) (mg/g DW) ^1^**						
**1**	515	449	287	Cyanidin 3-*O*-glucoside	0.02 ± 0.00	3.98 ± 0.08
**2**	500	595	433, 271	Pelargonidin 3,5-diglucoside	<LOQ	1.55 ± 0.03
**3**	502	433	271	Pelargonidin 3-*O*-glucoside	29.30 ± 0.59	266.76 ± 5.34
**4**	505	681	271	Pg 3-malonyldiglucoside	<LOQ	1.40 ± 0.03
**5**	507	519	271	Pg 3-malonylglucoside	5.20 ± 0.10	59.50 ± 1.19
**6**	505	475	271	Pg 3-acetylglucoside	<LOQ	0.87 ± 0.02
Total					34.52	334.06
**Peak Number**	***λ*_max_ (nm)**	**[M–H]^−^ (*m*/*z*)**	**MS ^2^**	**Tentative Identification**	**Whole Methanolic Extract**	**Anthocyanin-Enriched Fraction**
**Ellagic Acid Derivatives (Expressed as Ellagic Acid) (mg/g DW) ^1^**						
**7**	273	935, 467	633, 391, 301	Galloyl-bis- hexahydroxydipenoyl HHDP-glucose isomer	0.40 ± 0.03	8.07 ± 0.56
**8**	273	935, 447	301	Galloyl-bis-HHDP-glucose isomer	0.80 ± 0.06	21.17 ± 1.48
**9**	343	949, 477	779, 447, 301	Possible galloyl-HHDP-dehydrohexahydroxydiphenic acid-hexose	0.04 ± 0.00	2.14 ± 0.15
**10**	250/367	447	301	Ellagic acid deoxyhexoside	<LOQ	1.82 ± 0.13
**11**	250/366	301	284, 256	Ellagic acid	0.50 ± 0.04	11.40 ± 0.80
Total					1.74	44.60
**Flavonols/Dihydroflavonols (Expressed as, Quercetin or Kaempferol Glucoside) (mg/g DW) ^1^**						
**12**	352	477	301	Quercetin glucuronide	0.06 ± 0.00	2.98 ± 0.15
**13**	347	461	447, 285	Kaempferol glucuronide	0.10 ± 0.01	3.89 ± 0.19
**14**	347	489	285	Kaempferol acyl glucoside	0.10 ± 0.01	2.18 ± 0.11
Total					0.26	9.05

^1^ mg/g DW: mg of representative compound/g of the dried weight (DW) of the fraction.

**Table 3:** Comparison between strawberry fractions when applied at the same concentrations.

Parameters *	10 µg/mL			50 µg/mL		
	Whole Methanolic Extract	Anthocyanin-Enriched Fraction	Difference (%)	Whole Methanolic Extract	Anthocyanin-Enriched Fraction	Difference (%)
**Triglyceride Content**	0.75 ± 0.04 ^a^	0.63 ± 0.03 ^b^	12	0.63 ± 0.03 ^a^	0.46 ± 0.02 ^b^	17
**Low Density Lipoproteins-Cholesterol Content**	0.63 ± 0.03 ^a^	0.45 ± 0.02 ^b^	18	0.41 ± 0.02 ^a^	0.19 ± 0.01 ^b^	22
**Lipid Peroxidation**	0.63 ± 0.05 ^a^	0.29 ± 0.02 ^b^	34	0.42 ± 0.07 ^a^	0.18 ± 0.11 ^b^	24
**Intracellular Reactive Oxygen Species Production**	0.43 ± 0.04 ^a^	0.30 ± 0.03 ^b^	13	0.43 ± 0.03 ^a^	0.20 ± 0.01 ^b^	23
**Superoxide Dismutase Activity**	1.02 ± 0.01 ^a^	1.07 ± 0.04 ^a^	0.5	1.14 ± 0.03 ^a^	1.14 ± 0.02 ^a^	0
**Catalase Activity**	1.07 ± 0.04 ^b^	2.01 ± 0.18 ^a^	94	2.22 ± 0.14 ^b^	4.21 ± 0.41 ^a^	199

* Expressed as the fold increase with respect to the control. Different superscript letters for each evaluated parameter and corresponding with the same concentration indicate significant differences (*p* < 0.05) between strawberry fractions.
